# Immune Cell-Specific and Isoform-Selective Regulation of CD44 in Pancreatic Ductal Adenocarcinoma Links Lymph Node Variant Loss and Exosomal CD44 to Clinical Outcome in Pancreatic Ductal Adenocarcinoma

**DOI:** 10.3390/cells15050411

**Published:** 2026-02-27

**Authors:** Alara Karabiber, Yong Zhou, Anke Mittelstädt, Frederik Johannes Hansen, Melanie Litau, Isabelle Kuchenreuther, Johanne Mazurie, Finn Niklas Clausen, Sebastian Klöckner, Franziska Czubayko, Nadine Weisel, Bettina Klösch, Talida Andert-Veres, Stefanie Kröber, Susanne Merkel, Andreas R. R. Weiss, Maximilian Brunner, Christian Krautz, Robert Grützmann, Georg F. Weber, Paul David

**Affiliations:** 1 Department of Surgery, University Hospital Erlangen, Friedrich-Alexander University Erlangen-Nürnberg, 91054 Erlangen, Germany; alara.karabiber@uk-erlangen.de (A.K.); yong.zhou@extern.uk-erlangen.de (Y.Z.); anke.mittelstaedt@uk-erlangen.de (A.M.); frederik.hansen@ruhr-uni-bochum.de (F.J.H.); melanie.litau@uk-erlangen.de (M.L.); isi.s.kuchenreuther@fau.de (I.K.); johanne.mazurie@fau.de (J.M.); finn.clausen@fau.de (F.N.C.); sebastian.kloeckner@fau.de (S.K.); franziska.czubayko@uk-erlangen.de (F.C.); nadine.weisel@uk-erlangen.de (N.W.); bettina.kloesch@uk-erlangen.de (B.K.); susanne.merkel@uk-erlangen.de (S.M.); andreas.weiss@uk-erlangen.de (A.R.R.W.); maximilian.brunner@uk-erlangen.de (M.B.); christian.krautz@uk-erlangen.de (C.K.); robert.gruetzmann@uk-erlangen.de (R.G.); 2 Department of Anaesthesiology, University Hospital Erlangen, Friedrich-Alexander University Erlangen-Nürnberg, 91054 Erlangen, Germany; talidamarina.andert-veres@uk-erlangen.de (T.A.-V.); stefanie.kroeber@uk-erlangen.de (S.K.)

**Keywords:** CD44 variants, lymph nodes, exosomes, PDAC, immune checkpoints, metastasis, therapeutic resistance

## Abstract

Pancreatic ductal adenocarcinoma (PDAC) is characterized by immune cell dysfunction and poor prognosis. CD44, a cell surface glycoprotein with multiple splice variants, has been implicated in tumor progression, but its compartment-specific roles in PDAC remain unclear. CD44 standard and variant isoform expression was analyzed in patient-derived lymph nodes (LNs) by quantitative PCR. Immune cell-specific CD44 expression was assessed by flow cytometry in LNs and peripheral blood. Soluble and exosome-associated CD44 (exo-CD44) were measured in plasma. Clinical associations and survival analyses were performed. Transcriptomic, immune infiltration, immune checkpoint, and drug sensitivity analyses were conducted using TCGA-PAAD and pharmacogenomic datasets. CD44 standard isoform expression was unchanged in PDAC LNs, whereas multiple CD44 variant isoforms (v4–v10) were significantly reduced and associated with metastatic disease and poor survival, particularly CD44v5, v6, v7, and v10. CD44 expression was enriched in CD45^+^ immune cells, with highest levels in CD4^+^ T cells in both LNs and blood. Soluble CD44 levels showed no clinical associations. In contrast, exo-CD44 levels were reduced overall in PDAC but increased in patients with distant metastasis, positive resection margins, systemic inflammation, and reduced survival. High CD44 expression was associated with advanced disease, immune cell infiltration, immune checkpoint gene expression, reduced sensitivity to gemcitabine, paclitaxel, rapamycin, and FMK, and distinct CTLA4/PD-L1 checkpoint profiles. CD44 exhibits compartment-specific regulation in PDAC, linking immune remodeling, exosome signaling, and therapeutic resistance to adverse clinical outcome.

## 1. Introduction

Pancreatic ductal adenocarcinoma (PDAC) remains one of the deadliest malignancies, characterized by early dissemination, profound immune dysregulation, and limited biomarkers that reliably reflect disease progression or patient outcome [1,2]. Lymph nodes (LNs) are central hubs for antitumor immune responses and metastatic spread, yet molecular alterations within LN immune populations during PDAC progression remain poorly understood [3,4,5,6].

CD44 is a multifunctional transmembrane glycoprotein involved in cell adhesion, migration, lymphocyte activation, and tumor progression [7,8,9]. Alternative splicing of the CD44 gene generates multiple variant isoforms (CD44v), which have been implicated in cancer stemness, immune regulation, metastasis, and therapy resistance [10,11]. While the standard isoform (CD44s) is ubiquitously expressed, CD44 variants confer context-dependent functional specificity [12,13]. In PDAC, CD44 expression has been associated with aggressive tumor behavior; however, most studies have focused on tumor tissue rather than immune compartments such as lymph nodes or circulating blood [9,14].

Beyond membrane-bound CD44, soluble CD44 and exosome-associated CD44 (exo-CD44) represent additional layers of regulation [15,16,17]. Exosomes are increasingly recognized as mediators of tumor–immune crosstalk and systemic inflammation, and their cargo may reflect disease state more accurately than soluble plasma proteins [18,19,20,21]. However, the relative contributions and clinical relevance of CD44 splice variants, soluble CD44, and exosomal CD44 in PDAC have not been systematically evaluated.

In this study, we comprehensively analyzed CD44 isoform expression in lymph nodes, peripheral blood immune subsets, plasma, and circulating exosomes from PDAC patients and clinical control subjects. By integrating immune cell phenotyping, exosome profiling, and transcriptomic analyses, we aimed to determine whether CD44 dysregulation in PDAC primarily reflects tumor-cell expression or immune cell-specific remodeling. Our findings demonstrate isoform-selective and immune cell-restricted CD44 regulation in lymphoid tissues, reveal divergent exosomal CD44 dynamics associated with metastatic disease and systemic inflammation, and establish CD44 variants and exo-CD44 as clinically relevant indicators of immune dysfunction and prognosis in PDAC.

## 2. Materials and Methods

### 2.1. Sample Collection

For this study, we used PDAC patients (case group) and cholecystectomy and hernia repair patients (clinical control group) that underwent surgery at the surgical department of the University Hospital Erlangen, Germany, between 2020 and 2024. All patients were aged over 40 years and male patients were slightly more than females (35 vs. 30). For the study group consisting of 65 patients, patients had to undergo surgery for PDAC. For the control group that composed of 10 patients, patients were required to have a negative patient history of any form of malignancies (Table 1, Table 2 and Table 3).

All patients were evaluated and cleared by one of the medical doctors listed among the authors list and have provided written consent to the use of their body samples like blood, lymph node and pancreas tissue as well as both their medical history and data for research purposes. This study was conducted in accordance with the Declaration of Helsinki and approved by the Institutional Review Board of the University Hospital Erlangen (No. 180_19 B, 14 June 2019).

### 2.2. Sample Processing

Peripheral blood—preoperative blood samples were collected after the patients signed our consent form and drawn into two 7.5 mL EDTA tubes (Cat-No. 04.1921.001, Sarstedt, Nürnbrecht, Germany). To isolate the plasma, the tubes were centrifuged for 10 min at speed 350 × 18.9 *g* without brakes. Then, 45 mL of erythrocyte lysis buffer (Cat-No. 555899, BD Bioscience, Franklin Lakes, NJ, USA) was added to 5 mL cells and left at room temperature for 15 min to incubate, before centrifuging again for 5 min at speed 350 × 18.9 *g* brake 4. The lysed cells were washed with PBS and resuspended with FACS buffer (1× PBS, 1% FCS, 0.5% BSA, 2 mM EDTA) for further intra- and extracellular staining.

Lymph nodes: During robotic, laparoscopic or open surgery, lymph node station 8a was isolated from PDAC patients and 12b from control patients. Half of the lymph node tissue was frozen at −80 °C for relative mRNA expression analysis, and the rest was mechanically manipulated through a fine mesh strainer of 40 µm with the plunger of a 10 mL syringe and washed with 1X PBS. After centrifugation, the pellet was suspended with FACS buffer.

### 2.3. Quantitative Polymerase Chain Reaction (qPCR)

Real-time PCR was performed as previously described [21]. Briefly, RNA was extracted from the whole tissue by RNeasy mini kit (Qiagen, Venlo, The Netherlands). Complementary DNA was reverse-transcribed from 1 µg total RNA with Moloney murine leukemia virus reverse transcriptase (Thermo Fisher Scientific, Waltham, MA, USA) using random hexamer oligonucleotides for priming (Thermo Fisher Scientific). The amplification was performed with a Biorad CFX-Connect Real-time-System (Thermo Fisher Scientific) using the SYBR Green (Eurogentec, Seraing, Belgium) or TaqMan (Thermo Fisher Scientific) detection system. Data were analyzed using the Bio-Rad CFX Manager v3.1 software (Hercules, CA, USA). The mRNA contents for CD44 and its variants were normalized to the glyceraldehyde 3-phosphate dehydrogenase (GAPDH) mRNA for human genes. Gene expression was quantified using the ∆∆Ct method. The expression level was arbitrarily set to one for one sample from the control group and the values for the other samples were calculated relatively to this reference. The primer sequence of the genes quantified are as follows: Gapdh forward: 5′-AACAGCGACACCCACTCCTC-3′, Gapdh reverse: 5′-CATACCAGGAAATGAGCTTGACAA-3′; CD44s forward: 5′ GGAGCAGCACTTCAGGAGGTTAC 3′, CD44s reverse: 5′ GGAATGTGTCTTGGTCTCTGGTAGC 3′, CD44v2 forward: 5′ ATCACCGACAGCACAGACAGAAT 3′, CD44v2 reverse: 5′ AACCATGAAAACCAATCCCAGG 3′, CD44v3 forward: 5′ TACGTCTTCAAATACCATCTCAGCA 3′, CD44v3 reverse: 5′ AATCTTCATCATCATCAATGCCTG 3′, CD44v4 forward: 5′ AACCACACCACGGGCTTTTG 3′, CD44v4 reverse: 5′ TCCTTGTGGTTGTCTGAAGTAGCA 3′, CD44v5 forward: 5′ TGCTTATGAAGGAAACTGGAAC 3′, CD44v5 reverse: 5′ TGTGCTTGTAGAATGTGGGGT 3′, CD44v6 forward: 5′ CCAGGCAACTCCTAGTAGTACAACG 3′, CD44v6 reverse: 5′ CGAATGGGAGTCTTCTTTGGGT 3′, CD55v7 forward: 5′ GCCTCAGCTCATACCAGCCATC 3′, CD44v7 reverse: 5′ TCCTTCTTCCTGCTTGATGACCT 3′, CD44v8 forward: 5′ TGGACTCCAGTCATAGTATAACGC 3′, CD44v8 reverse: 5′ GGTCCTGTCCTGTCCAAATC 3′, CD44v9 forward: 5′ AGCAGAGTAATTCTCAGAGC 3′, CD44v9 reverse: 5′ TGATGTCAGAGTAGAAGTTGTT 3′, CD44v10 forward: 5′ CCTCTCATTACCCACACACG 3′, CD44v10 reverse: 5′ CAGTAACTCCAAAGGACCCA 3′.

### 2.4. Flow Cytometry Analysis

For peripheral blood analysis, samples were lysed using a lysis buffer. The cells were then incubated with an antibody cocktail for 30 min at 4 °C. Following incubation, the cells were washed using FACS buffer (1× PBS, 1% FCS, 0.5% BSA, 2 mM EDTA) and were prepared for acquisition. The antibodies used were as follows: Anti-CD68-FITC (Cat-No. 333805, BioLegend, San Diego, CA, USA), anti-CD44-PE (Cat-No. 749146, BD Bioscience, Franklin Lakes, NJ, USA), anti-CD8a-PE Dazzle (Cat-No. 562311, BD Bioscience, Franklin Lakes, NJ, USA), anti-CD4-PerCP-Cy5.5 (Cat-No. 560650, BD Bioscience, Franklin Lakes, NJ, USA), anti-HLADR-BUV395 (Cat-No. 564040, BD Bioscience, Franklin Lakes, NJ, USA), anti-CD14-BUV737 (Cat-No. 612763, BD Bioscience, Franklin Lakes, NJ, USA), anti-CD56-BV421 (Cat-No. 362551, BioLegend, San Diego, CA, USA), anti-CD3-BV510 (Cat-No. 563109, BD Bioscience, Franklin Lakes, NJ, USA), anti-CD20-BV650 (Cat-No. 563780, BD Bioscience, Franklin Lakes, NJ, USA), anti-CD11c-BV711 (Cat-No. 563130, BD Bioscience, Franklin Lakes, NJ, USA), anti-CD45-BV786 (Cat-No. 563716, BD Bioscience, Franklin Lakes, NJ, USA). The data was sampled by using the BD Celesta flow cytometer (BD Bioscience, Franklin Lakes, NJ, USA) and the BD FACSDiva™ software v8.0.1.1 and analyzed with FlowJo 10.9.0 (FlowJo LLC, Ashland, OR, USA).

### 2.5. Enzyme-Linked Immunosorbent Assay (ELISA) of CD44

The Human CD44 DuoSet ELISA was performed according to the manufacturer’s (R&D Systems, Minneapolis, MN, USA; DY7045-05) protocol and instructions. Plasma samples of PDAC patients and controls were diluted 1:10 and 100 µL of the dilution was added into a 96 well. The microplate was precoated with a monoclonal antibody specific for human CD44. After pipetting, the plate was left at room temperature for two hours and transferred into the refrigerator at 4 °C to further incubate overnight. The next day, the plate was washed three times and an enzyme-linked polyclonal antibody specific for human CD44 was added. After repeating the washing step, the substrate solution Streptavidin–Horseradish peroxidase was added, turning the wells blue in proportion to the amount of antigen present in the samples. The substrate was left for 30 min at room temperature before stopping further development by adding 2 N H_2_SO_4_. The color intensity was measured by the plate reader SpectraMax M3 by Molecular Devices (San Jose, CA, USA) at 450 nm.

### 2.6. Exosome Isolation, Quantification, NTA, Immunogold Labeling and Electron Microscopy

Exosome isolation and characterization in the current study were performed following the established protocols described by [20,21].

### 2.7. MACSPlex Exosome Assay

Exosome analysis was performed using the MACSPlex Human Exosome Kit (Cat. No. 130-108-813, Miltenyi Biotec, Bergisch Gladbach, Germany), as previously described [20,21]. The assay utilizes 4–8 µm polystyrene beads, each uniquely encoded with defined ratios of phycoerythrin (PE) and fluorescein isothiocyanate (FITC), generating 39 bead populations distinguishable by flow cytometry. Each bead subset is coated with a specific capture antibody targeting an extracellular vesicle (EV) surface antigen (37 EV markers and two isotype controls). Plasma samples were incubated with the bead mixture overnight to allow binding of EVs to their respective capture beads. On the following day, EVs attached to beads were detected using an allophycocyanin (APC)-conjugated antibody cocktail against CD9, CD63, and CD81 (Figure S3). Samples were analyzed on a flow cytometer equipped with blue, red, violet, UV, and yellow–green lasers. PBS was processed in parallel to determine background signal. For data processing, the median fluorescence intensity (MFI) of each bead population was background-corrected by subtracting the MFI of the corresponding PBS control. Values were then normalized to a sample-specific normalization factor, calculated as the mean MFI of the tetraspanins CD9, CD63, and CD81. The resulting normalized intensities represent the relative abundance of each EV surface marker within the sample.

### 2.8. Bioinformatic Analyses of CD44 Function in Pancreatic Cancer

To investigate the functional significance of CD44 expression in pancreatic cancer, we downloaded and processed RNA-sequencing data and corresponding clinical information for 185 pancreatic ductal adenocarcinoma (PDAC) samples from The Cancer Genome Atlas Pancreatic Adenocarcinoma cohort (TCGA-PAAD; https://portal.gdc.cancer.gov/; accessed on 5 September 2025). After quality control and pre-processing as described in our previous publication, 179 PDAC samples were retained for downstream analyses [22].

Prognostic nomograms were constructed using the “rms” R package (v7.0.0) to evaluate the predictive value of CD44 in combination with clinical variables. CD44 co-expression networks were assessed using Pearson correlation analysis, applying a significance threshold of |R| > 0.65 and *p* < 0.001 to identify robustly associated genes. To estimate the immune and stromal components of the tumor microenvironment (TME), CD44 expression was integrated with TME scoring algorithms using the “estimate” (v1.0.13) and “limma” (v3.62.2) R packages (Becht et al., 2016) [23]. Immune cell infiltration patterns were quantified with the CIBERSORT algorithm, providing insights into CD44-associated immune composition within the PDAC microenvironment.

Given the relevance of immune checkpoint blockade (ICB) in PDAC, CD44 expression was further correlated with 34 ICB-related genes as described by Tang et al. (2021) [24] and Xu et al. (2021) [25], using Pearson correlation coefficients to identify immune regulatory associations. To explore potential therapeutic vulnerabilities linked to high CD44 expression, drug sensitivity prediction was performed using the “pRRophetic” package (v0.5), which estimates chemotherapeutic response based on gene expression signatures. Data on immunotherapy response were obtained from The Cancer Immunome Atlas (TCIA) (https://tcia.at/home; accessed on 5 September 2025), containing response profiles for 178 samples treated with immune-based therapies. Differences in predicted immunotherapy sensitivity were visualized using “ggpubr” (v0.6.2) built on ggplot2.

### 2.9. Statistical Analysis

The primary program used to determine statistics was GraphPad Prism (Version 9.5.1 (733) for Windows, GraphPad Software, Boston, MA, USA). For the measurement comparison of two groups, unpaired *t*-test with Welch’s correction was applied. Normality was assessed using the Shapiro–Wilk test. For counting the data and determination of the independence of two categorical variables. Pearson Chi square was used. Correlation analysis was performed using linear regression; two-tailed *p*-values as well as R^2^ values were conducted. Survival was analyzed using Kaplan–Meier curves and the log-rank (Mantel–Cox) test, with hazard ratios (HRs) and 95% confidence interval (95% CIs), by dividing patients into low- and high-expression groups of Exo-CD44 or CD44 and its variants in lymph node and pancreas tissue. Due to the relatively small sample size, we did not perform a multivariate analysis to evaluate CD44 as an independent prognostic factor. IBM SPSS software (SPSS-Software, Version: 28.0.0.0 (190), Armonk, NY, USA) was used to determine odds ratios. Statistical significance was set at *p* < 0.05.

## 3. Results

### 3.1. CD44 Standard Isoform Is Unchanged in PDAC Lymph Nodes, While CD44 Variant Isoforms Are Significantly Reduced

**Figure 1:**
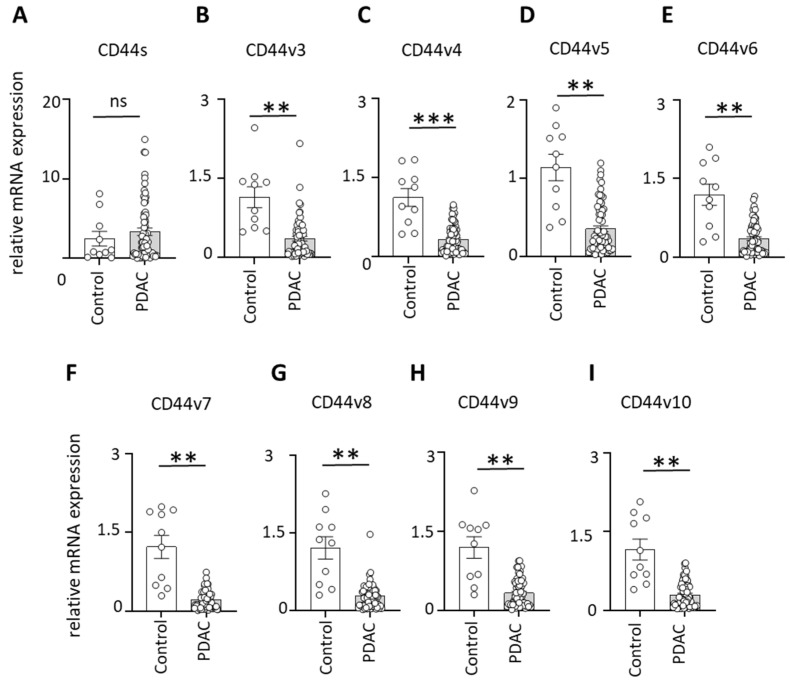
CD44 standard and variant isoform expression in lymph nodes of PDAC patients. (**A**) Relative mRNA expression of CD44 standard isoform (CD44s) between PDAC patients (n = 65) and clinical controls (n = 11). (**B**–**I**) CD44 variant isoforms v3, v4, v5, v6, v7, v8, v9 and v10 in control versus PDAC patients; *** = *p* < 0.001; ** = *p* < 0.01; ns = not significant.

Quantitative PCR analysis of lymph node (LN) samples revealed no significant difference in CD44 standard isoform (CD44s) expression between PDAC patients and clinical control subjects (Figure 1A). In contrast, multiple CD44 variant isoforms were markedly reduced in PDAC LNs.

Specifically, PDAC patients exhibited significantly decreased expression of CD44v3, v4, v5, v6, v7, v8, v9, and v10 compared to controls (Figure 1B–I). These findings indicate that PDAC is associated with selective downregulation of CD44 splice variants rather than global suppression of CD44 expression within lymphoid tissues.

### 3.2. Loss of CD44 Variant Expression in Lymph Nodes Is Associated with Metastasis and Poor Survival in PDAC

**Figure 2:**
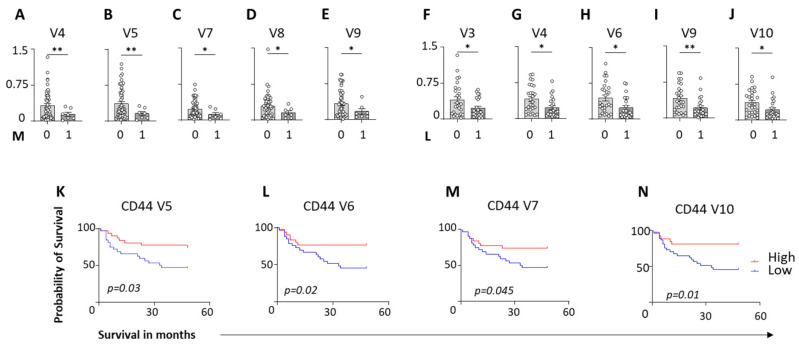
Loss of CD44 variant expression correlates with metastatic progression and poor prognosis in PDAC. (**A**–**E**) Comparative analysis of CD44 variant expression by metastatic status. qPCR data shows a significant reduction in lymph node (LN) expression of CD44v4, v5, v7, v8, and v9 in patients with distant metastasis (n = 7) compared to localized metastasis (n = 51) in pancreatic ductal adenocarcinoma (PDAC) patients. (**F**–**J**) Association of CD44 variants with lymphatic invasion. Further stratification reveals significant downregulation of CD44v3, v4, v6, v9, and v10 in patients with confirmed lymphatic invasion (L1) (n = 27) disease versus lymphatic invasion-negative (L0) (n = 23) disease. (**K**–**N**) Kaplan–Meier survival curves stratified by CD44 variant expression. PDAC patients with low LN (n = 32) expression of CD44v5, v6, v7, and v10 (blue lines) exhibit significantly reduced overall survival (OS) compared to patients with high expression (n = 33) (red lines). * = *p* < 0.05; ** = *p* < 0.01.

Stratification of PDAC patients by metastatic status revealed a strong association between disease dissemination and reduced CD44 variant expression in lymph nodes. Patients with metastatic PDAC exhibited significantly lower expression of multiple CD44 variants, including CD44v4, v5, v7, v8, and v9 compared to tumor-free LNs (Figure 2A–E). Furthermore, patients with confirmed lymphatic invasion status showed additional reductions in CD44v3, v4, v6, v9, and v10, indicating progressive loss of CD44 variant expression with increasing metastatic burden (Figure 2F–J).

Survival analysis demonstrated that reduced expression of specific CD44 variants in lymph nodes was associated with adverse clinical outcomes. PDAC patients with low LN expression of CD44v5, CD44v6, CD44v7, and CD44v10 experienced significantly shorter overall survival compared to patients with high expression of these variants (Figure 2K–N). No difference was observed in local tumor size and extent (pT), nodal stage (pN), perineural invasion (Pn), venous invasion (V), grading (G), and UICC staging (Figures S1–S3). To understand the neoadjuvant treatment effects on CD44 and to evaluate the influence of neoadjuvant therapy on CD44 isoform regulation, lymph node CD44 variant expression was stratified according to treatment status (Table 2). A tendency toward increased CD44v7 expression (*p = 0.052*) was observed in patients who received neoadjuvant therapy compared with untreated patients, although this difference did not reach statistical significance. In contrast, CD44v8 expression was significantly altered in the neoadjuvant-treated group. Importantly, the overall pattern of CD44 variant downregulation in PDAC lymph nodes particularly for CD44v4–v10 remained evident after the exclusion of neoadjuvant-treated patients (Figure S3). Furthermore, the associations between low CD44v5/v6/v7/v10 expression and reduced overall survival persisted in treatment-stratified analyses. These findings suggest that while specific CD44 variants (notably v8) may be modulated by systemic therapy, the broader pattern of CD44 variant loss in PDAC lymph nodes is not solely attributable to neoadjuvant treatment effects.

Collectively, these findings suggest that a loss of CD44 variant expression in lymph nodes reflects impaired immune surveillance or lymph node remodeling during PDAC progression. Hence, CD44 variant levels in lymphoid tissues may serve as prognostic markers reflecting immune competence in PDAC.

### 3.3. Immune Cell-Specific CD44 Expression in Lymph Nodes and Blood Supports Compartment-Specific CD44 Variant Regulation in PDAC

**Figure 3:**
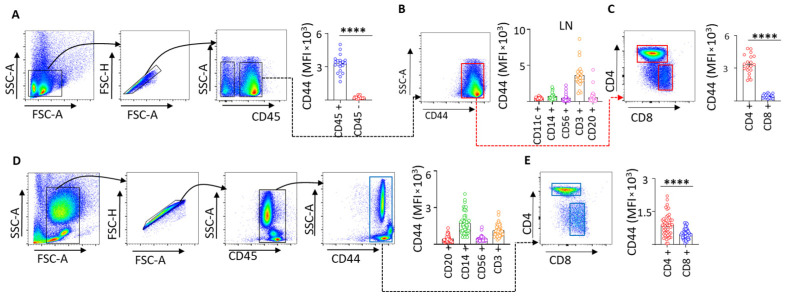
FACS gating strategy and CD44 expression in immune cell subsets from PDAC lymph nodes and peripheral blood. Flow cytometry was used to assess CD44 expression across immune cell subsets in PDAC lymph nodes and peripheral blood. (**A**,**B**) Gating strategy and representative plots showing higher CD44 expression in CD45^+^ immune cells compared to CD45^−^ cells. (**C**) CD4^+^ T cells exhibited higher CD44 expression than CD8^+^ T cells within lymph nodes (n = 25). (**D**,**E**) Peripheral blood (n = 48) analysis of CD44 expression in CD14^+^ monocytes, followed by CD3^+^ T cells, with CD4^+^ T cells. **** = *p* < 0.0001.

Flow cytometric analyses demonstrated that CD44 expression in PDAC lymph nodes is predominantly localized to CD45^+^ immune cells (Figure 3A), with the highest expression observed in CD3^+^ T lymphocytes (Figure 3B), particularly CD4^+^ T cells, compared with CD8^+^ T cells (Figure 3C). A consistent pattern was observed in preoperative peripheral blood, where CD14^+^ monocytes and CD4^+^ T cells exhibited the highest CD44 expression (Figure 3D,E). These data indicate that CD44 expression in PDAC is largely immune-associated and conserved across lymphoid and circulating compartments.

When integrated with our lymph node transcriptomic analyses, these findings suggest that the selective loss of CD44 variant isoforms (CD44v) observed in PDAC lymph nodes occurs within immune-enriched compartments rather than reflecting a global reduction in CD44 expression. Given the preferential expression of CD44 in CD4^+^ T cells, loss of CD44 variant isoforms may reflect altered immune cell activation, trafficking, or retention within lymph nodes during PDAC progression. This interpretation is further supported by the association between reduced CD44 variant expression, metastatic disease, and poor survival.

Together, these data support a model in which CD44 variant dysregulation within immune cell-rich lymphoid tissues contributes to impaired immune surveillance in PDAC, while preserved or elevated immune- and exosome-associated CD44 in other compartments reflects advanced disease and immune remodeling. This compartment-specific regulation of CD44 provides a mechanistic framework linking immune cell biology, lymph node remodeling, and disease progression in pancreatic cancer.

### 3.4. Soluble and Exosome-Associated CD44 Display Distinct Clinical Associations in PDAC

**Figure 4:**
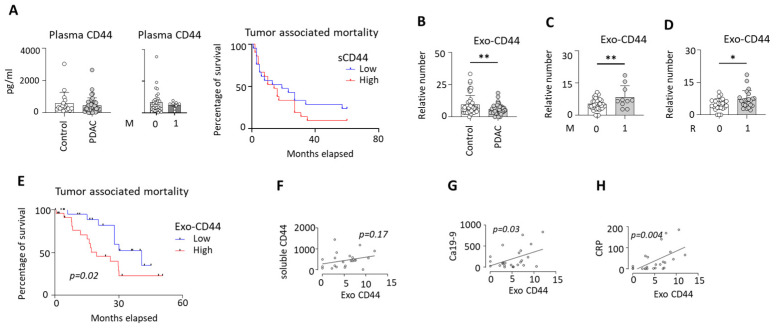
Distinct clinical associations of soluble CD44 and exosome-associated CD44 in PDAC. (**A**) Soluble CD44 levels in PDAC patients and controls, disease stage and survival. (**B**–**D**) Exosome-associated CD44 (exo-CD44) in PDAC and control; PDAC patients with distant metastasis and positive resection margins. (**E**) Kaplan–Meier plot denoting the survival difference between low and high exo-CD44. (**F**–**H**) Exo-CD44 correlation with soluble CD44, CA19-9 and CRP. * = *p* < 0.05; ** = *p* < 0.01.

Measurement of soluble CD44 levels in plasma revealed no significant differences between PDAC patients and clinical control subjects. Furthermore, soluble CD44 levels did not correlate with disease stage, metastatic status, or overall survival, indicating limited utility of soluble CD44 as a biomarker in PDAC (Figure 4A).

In contrast, the analysis of exosome-associated CD44 (exo-CD44) using the MACSPlex exosome assay demonstrated significant alterations associated with disease progression. Overall, PDAC patients exhibited significantly lower exo-CD44 levels compared to clinical controls (Figure 4B). However, within the PDAC cohort, exo-CD44 levels were significantly increased in patients with distant metastasis compared to those with localized disease (Figure 4C). Similarly, patients with positive resection margins (R1) showed higher exo-CD44 levels than those with negative margins (R0), linking elevated exo-CD44 to aggressive tumor behavior (Figure 4D). Survival analysis further revealed that PDAC patients with high exo-CD44 levels experienced significantly reduced overall survival compared to patients with low exo-CD44 levels (Figure 4E).

Notably, exo-CD44 levels did not correlate with soluble CD44 concentrations, suggesting that circulating exosomal and soluble CD44 are regulated by distinct biological mechanisms (Figure 4F). Importantly, exo-CD44 levels showed significant positive correlations with CA19-9 and C-reactive protein (CRP) and we did not observe any correlations with age, gender, bilirubin and leucocytes count, implicating exo-CD44 as a marker of systemic inflammation and tumor burden in PDAC (Figure 4G,H; Figures S3 and S4). Table 4 provides details on the characteristic features of the study population, categorized based on exosomal CD44 absolute numbers in PDAC patients.

### 3.5. CD44 Expression Is Associated with Survival, Clinicopathological Features, and Immune Signatures in PDAC

**Figure 5:**
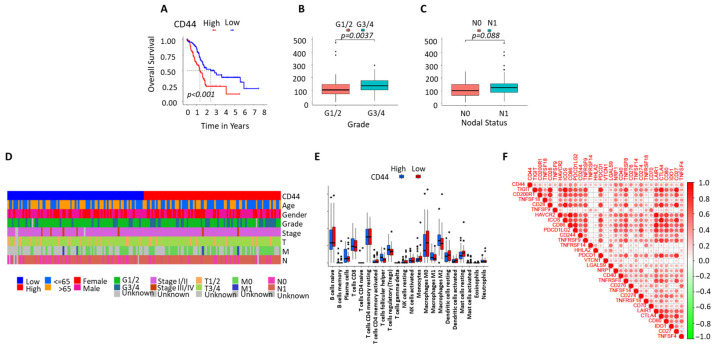
Association of CD44 expression with survival, clinicopathological features, and immune signatures in PDAC. (**A**) Kaplan–Meier overall survival analysis of PDAC patients stratified by CD44 expression (*p* < 0.001; log-rank test). (**B**) Comparison of CD44 expression according to tumor grade. Higher CD44 expression was observed in high-grade tumors (G3/4) compared with low-grade tumors (G1/2) (*p* = 0.0037; Wilcoxon rank-sum test). (**C**) CD44 expression according to lymph node status. A trend toward increased CD44 expression was observed in patients with lymph node involvement (N1) compared with node-negative patients (N0) (*p* = 0.088). (**D**) Heatmap summarizing the association between CD44 expression and clinicopathological parameters, including tumor stage and nodal status. (**E**) Immune cell infiltration analysis comparing CD44-high and CD44-low tumors. Elevated CD44 expression was associated with increased infiltration of CD4^+^ T cells, macrophages, monocytes, and dendritic cells. (**F**) Correlation analysis between CD44 expression and immune checkpoint and immune regulatory genes, including PDCD1 (PD-1), CD274 (PD-L1), PDCD1LG2, CTLA4, TIGIT, ICOS, LAG3, and TNFRSF family members.

Kaplan–Meier analysis showed that PDAC patients with high CD44 expression had significantly reduced overall survival compared with patients with low CD44 expression (*p* < 0.001) (Figure 5A). The clinicopathological characteristics grouped according to the median CD44 expression are shown in Supplementary Table S1. Higher CD44 expression was significantly associated with advanced tumor grade (G3/4 versus G1/2; *p* = 0.0037) (Figure 5B). A trend toward increased CD44 expression was observed in patients with lymph node involvement (N1 versus N0; *p* = 0.088) (Figure 5C).

Annotation of clinicopathological parameters demonstrated that high CD44 expression clustered with advanced tumor stage and nodal metastasis, while no clear association was observed with age or sex (Figure 5D). These findings are consistent with our experimental lymph node analyses, showing altered CD44 regulation in metastatic PDAC.

Immune cell profiling revealed that high CD44 expression was associated with differences in immune cell infiltration, including increased levels of CD4^+^ T cells, macrophages, monocytes, and dendritic cells (Figure 5E). These data align with our flow cytometry results demonstrating enrichment of CD44 expression in CD45^+^ immune cells, particularly CD4^+^ T cells, in PDAC lymph nodes and peripheral blood.

Correlation analysis demonstrated positive associations between CD44 expression, multiple immune checkpoints, and immune regulatory molecules, including PDCD1 (PD-1), CD274 (PD-L1), PDCD1LG2, CTLA4, TIGIT, ICOS, LAG3, and TNFRSF family members (Figure 5F). These associations are consistent with our exosome analyses, showing that exosome-associated CD44 levels are linked to advanced disease and poor survival, whereas soluble CD44 levels in plasma showed no association with clinical outcome.

### 3.6. CD44 Co-Expression Networks and Immune-Related Gene Correlations in PDAC (TCGA-PAAD)

**Figure 6:**
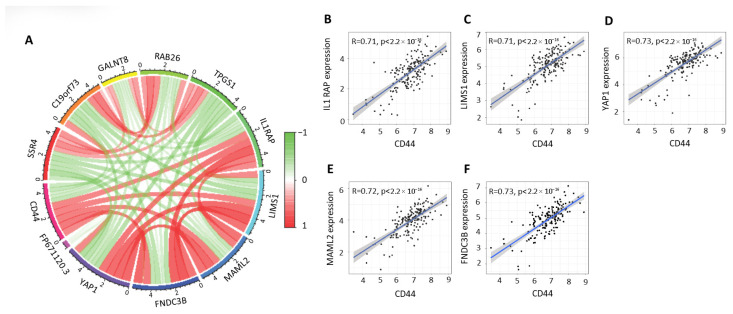
CD44 co-expression networks and immune-related gene correlations in PDAC (TCGA-PAAD). (**A**) Circos plot depicting the CD44-centered co-expression network in the TCGA-PAAD dataset. CD44 shows coordinated positive (green) and negative (red) correlations with genes involved in immune regulation, extracellular matrix organization, and signaling pathways, including IL1RAP, TPGS1, RAB26, GALNT8, FNDC3B, YAP1, and MAML2. (**B**–**F**) Scatter plots showing strong positive correlations between CD44 expression and representative immune-related genes within TCGA-PAAD (R = 0.71–0.73, *p* < 2.2 × 10^−16^). Each panel displays linear regression with 95% confidence intervals.

Transcriptomic analyses were performed using publicly available pancreatic ductal adenocarcinoma datasets from the Cancer Genome Atlas Pancreatic Adenocarcinoma (TCGA-PAAD) cohort. Co-expression network analysis revealed that CD44 is embedded within a broad transcriptional module comprising genes involved in immune regulation, extracellular matrix remodeling, and tumor–immune interactions (Figure 6A). CD44 demonstrated coordinated positive and negative correlations with multiple genes, including IL1RAP, LIMS1, YAP1, MAML2 and FNDC3B, indicating that CD44 participates in structured gene expression programs relevant to PDAC biology.

Correlation analyses further demonstrated strong positive associations between CD44 expression and immune-related gene signatures within the TCGA-PAAD cohort (Figure 6B–F). CD44 expression showed robust correlations (R ≈ 0.71–0.73, *p* < 2.2 × 10^−16^) with genes associated with immune activation, immune cell trafficking, and inflammatory signaling, confirming a close relationship between CD44 expression and immune-related transcriptional activity in PDAC tumors.

These TCGA-PAAD-derived findings are consistent with our experimental analyses of patient-derived lymph nodes, in which CD44 expression was predominantly localized to CD45^+^ immune cells, particularly CD4^+^ T cells, and where selective loss of CD44 variant isoforms was associated with metastatic disease and reduced survival. Furthermore, the strong association of CD44 with immune-related gene networks aligns with our exosome profiling data showing that exosome-associated CD44 (exo-CD44), but not soluble CD44, correlates with advanced disease, systemic inflammation, and adverse clinical outcome.

Together, these data indicate that CD44 expression in PDAC reflects immune-associated transcriptional programs across tumor tissue, lymphoid compartments, and circulating exosomes, supporting a compartment-specific role for CD44 in cancer–immune system interactions.

### 3.7. High CD44 Expression Is Associated with Reduced Drug Sensitivity and Distinct Immune Checkpoint Profiles in PDAC

**Figure 7:**
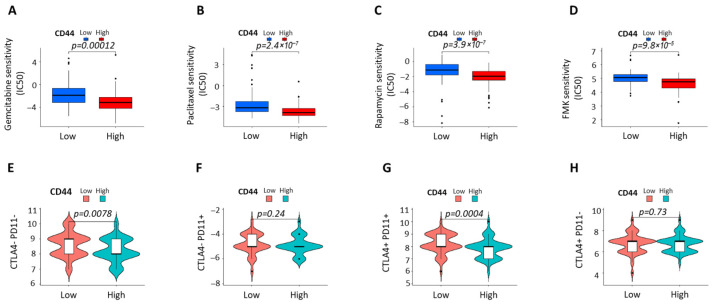
Association of CD44 expression with drug sensitivity and immune checkpoint profiles in PDAC. (**A**–**D**) Drug sensitivity analysis comparing CD44-high and CD44-low PDAC cases. CD44-high tumors exhibited significantly higher IC50 values for Gemcitabine, Paclitaxel, Rapamycin, and FMK, indicating reduced sensitivity to cytotoxic and targeted agents. Data are derived from pharmacogenomic analyses and are presented as median with interquartile range; statistical significance was determined using the Wilcoxon rank-sum test. (**E**–**H**) Immune checkpoint stratification of PDAC tumors based on combined CTLA4 and PD-L1 expression. CD44-high tumors displayed significantly lower proportions of CTLA4^−^PD-L1^−^ and CTLA4^+^PD-L1^+^ subgroups compared with CD44-low tumors. No significant differences were observed in the CTLA4^−^PD-L1^+^ or CTLA4^+^PD-L1^−^ subgroups. Statistical significance was assessed using the chi-square test.

Drug sensitivity analysis showed that PDAC cases with high CD44 expression exhibited significantly reduced sensitivity to Gemcitabine, Paclitaxel, Rapamycin, and FMK, as reflected by higher IC50 values compared with CD44-low cases (Figure 7A–D). These findings indicate an association between elevated CD44 expression and therapy-resistant phenotypes in PDAC.

Immune checkpoint stratification revealed distinct CTLA4/PD-L1 expression patterns according to CD44 status. CD44-high tumors displayed significantly lower proportions of CTLA4^−^PD-L1^−^ and CTLA4^+^PD-L1^+^ subgroups than CD44-low tumors, while no differences were observed in CTLA4^−^PD-L1^+^ or CTLA4^+^PD-L1^−^ groups (Figure 7E–H). This suggests selective modulation of coordinated immune checkpoint states rather than isolated regulation of CTLA4 or PD-L1.

## 4. Discussion

In this study, we provide a comprehensive, multi-compartment analysis of CD44 biology in pancreatic ductal adenocarcinoma (PDAC), integrating lymph node-based molecular profiling, immune cell-specific expression, circulating soluble and exosome-associated CD44, and transcriptomic and pharmacogenomic analyses. Our findings reveal a compartment-specific and isoform-specific regulation of CD44 that links immune remodeling, metastatic progression, therapeutic resistance, and clinical outcome in PDAC.

A key observation of our study is that the CD44 standard isoform (CD44s) remains unchanged in PDAC lymph nodes, whereas multiple CD44 variant (CD44v) isoforms are selectively and significantly reduced. This distinction is important, as CD44s and CD44v isoforms have divergent biological functions [13,26,27]. CD44v isoforms play key roles in immune cell trafficking, lymphocyte activation, and maintenance of tissue architecture [7]. Their selective loss in PDAC lymph nodes—particularly CD44v4–v10—suggests that PDAC progression is associated not with global CD44 suppression but with altered alternative splicing that may compromise lymph node immune competence [28,29,30].

The association between CD44 variant loss and metastatic disease further supports this concept. Patients with metastatic PDAC and confirmed lymph node involvement exhibited progressively reduced expression of multiple CD44 variants, indicating that CD44v depletion may reflect lymph node remodeling or impaired immune surveillance during tumor dissemination [31,32]. Importantly, low expression of CD44v5, CD44v6, CD44v7, and CD44v10 in lymph nodes was associated with significantly reduced overall survival, highlighting the prognostic relevance of CD44 variant expression within immune tissues rather than tumor tissue alone [7,14].

Flow cytometric analyses revealed that CD44 expression in PDAC lymph nodes is predominantly localized to CD45^+^ immune cells, with the highest expression observed in T lymphocytes, particularly CD4^+^ T cells. This pattern was conserved in peripheral blood, where CD14^+^ monocytes and CD4^+^ T cells showed the highest CD44 expression. These findings indicate that CD44 expression in PDAC is primarily immune-associated and suggest that alterations in CD44 variant expression may directly affect immune cell function, trafficking, or activation, both locally and systemically [33,34].

Our analysis of circulating CD44 further underscores the compartment-specific nature of CD44 regulation. Soluble CD44 levels in plasma did not differ between PDAC patients and controls and showed no association with disease stage, metastasis, or survival, indicating limited biomarker utility. In contrast, exosome-associated CD44 (exo-CD44) displayed a distinct and clinically meaningful pattern. While overall exo-CD44 levels were reduced in PDAC patients compared to controls, elevated exo-CD44 within the PDAC cohort was associated with distant metastasis, positive resection margins, systemic inflammation (CRP), tumor burden (CA19-9), and reduced survival. The lack of correlation between soluble and exosomal CD44 suggests that these pools are regulated by independent biological mechanisms, with exo-CD44 potentially reflecting active tumor–immune communication rather than passive shedding [35,36].

Transcriptomic analyses using the TCGA-PAAD cohort further contextualized our experimental findings [22]. CD44 was embedded within immune-related co-expression networks and strongly correlated with genes involved in immune activation, inflammatory signaling, and immune checkpoint regulation. Consistent with this, high CD44 expression was associated with increased immune cell infiltration, including CD4^+^ T cells, macrophages, monocytes, and dendritic cells, as well as elevated expression of multiple immune checkpoint molecules, including PD-1, PD-L1, CTLA4, TIGIT, ICOS, and LAG3 [37,38,39]. These data suggest that CD44-high tumors exist within an immune-infiltrated yet potentially immunosuppressed microenvironment.

Notably, immune checkpoint stratification revealed that CD44-high tumors exhibited reduced proportions of both CTLA4^−^PD-L1^−^ and CTLA4^+^PD-L1^+^ subgroups, without differences in discordant checkpoint states. This selective redistribution implies coordinated immune checkpoint regulation rather than simple upregulation or loss of individual inhibitory receptors and may reflect altered immune cell activation or exhaustion states linked to CD44-associated signaling [40,41,42].

Stratified analyses revealed that neoadjuvant therapy exerts selective effects on CD44 isoform regulation within lymph nodes. While CD44v7 showed a non-significant trend, CD44v8 was significantly altered in treated patients, consistent with reports that chemotherapy can induce CD44 upregulation or isoform switching, particularly in therapy-resistant or stem-like cancer cell populations [14,43]. CD44v8 has been linked to redox regulation and cytotoxic stress resistance, supporting the possibility that systemic therapy influences its expression. Importantly, however, the overall downregulation of CD44 variants (v4–v10) in PDAC lymph nodes and their association with survival persisted after exclusion of treated patients. These findings indicate that while specific variants may be modulated by therapy, the broader pattern of CD44 variant loss reflects tumor-driven immune remodeling rather than a treatment-induced artifact.

In parallel, pharmacogenomic analyses demonstrated that high CD44 expression is associated with reduced sensitivity to Gemcitabine, Paclitaxel, Rapamycin, and FMK, suggesting a link between CD44 expression and therapy resistance [44,45,46,47]. This finding aligns with the association between elevated exo-CD44 and poor survival and supports a model in which CD44 contributes to aggressive disease behavior through both immune modulation and treatment resistance.

Collectively, our data support a model in which CD44 plays distinct, compartment-specific roles in PDAC. Loss of CD44 variant expression in lymph nodes may impair immune surveillance and contribute to metastatic progression, while elevated immune- and exosome-associated CD44 reflects an immunologically active yet suppressed tumor microenvironment associated with systemic inflammation, therapeutic resistance, and poor outcome. These findings highlight CD44 not as a uniform biomarker but as a dynamic regulator of cancer–immune interactions whose functional impact depends on isoform usage and biological compartment.

Future studies will be required to define the mechanistic consequences of CD44 variant loss in lymphoid tissues and to determine whether targeting CD44-mediated immune and exosome signaling pathways could improve immune function or therapeutic response in PDAC. Nonetheless, our study provides a framework for understanding CD44 as a key integrator of immune regulation and disease progression in pancreatic cancer.

Limitations: First, the patient cohort size, particularly for subgroup analyses stratified by metastatic status and resection margin, was limited and may reduce statistical power for certain comparisons. Second, CD44 variant expression was assessed at the mRNA level in lymph node tissues; corresponding protein-level validation for individual CD44 variant isoforms was not feasible due to antibody limitations. Finally, bioinformatic analyses were derived from publicly available datasets and may not fully recapitulate patient-specific tumor heterogeneity. Future studies incorporating larger cohorts, functional immune assays, and longitudinal sampling will be required to validate and mechanistically extend these findings.

## 5. Conclusions

CD44 shows distinct, compartment-specific behavior in pancreatic cancer, with loss of CD44 variants in lymph nodes marking immune disruption and metastatic progression, while elevated exosomal and tumor CD44 identify aggressive, immune-modulated disease. Soluble CD44 has no clinical value, whereas exo-CD44 and tumor CD44 strongly associate with inflammation, poor survival, and predicted chemoresistance. These find-ings establish CD44 as a context-dependent biomarker and potential therapeutic target in PDAC.

## Figures and Tables

**Table 1:** Characteristic features of clinical control patients (qPCR).

	Clinical Control Patients
Number	11
Mean age (in years [range])	61.83 (38–84)
Sex (male: female)	(4:7)
Cholecystectomy (CHE)	11

**Table 2:** Characteristic features of PDAC patient cohort (qPCR).

		PDAC Patients
Number		65
Mean age (in years [range])		68.43 (42–90)
Sex (male: female)		(34:31)
pT category	pT1	6
	pT2	21
	pT3	16
	pT4	1
	Unknown/Unresectable	21
pN category	pN0	12
	pN1,2	31
	Unknown/Unresectable	22
Venous invasion	V0	37
	V1	5
	Unknown/Unresectable	23
Lymphatic invasion	L0	23
	L1	21
	Unknown/Unresectable	21
Perineural invasion	Pn0	12
	Pn1	31
	Unknown/Unresectable	22
R classification	R0	46
	R1	3
	R2/inoperable	16
Grading	G1	3
	G2	9
	G3	35
	Unknown/Unresectable	18
Distant metastasis	No	56
	Yes	9
UICC	I	14
	II	23
	III	14
	IV	9
	Unknown/Unresectable	5
Neoadjuvant treatment	No	52
	Yes	13

**Table 3:** Characteristic features of clinical control and PDAC patient cohort (exosomes).

	Clinical Control Patients	PDAC
Number	51	49
Mean Age (in years [range])	61.3 (38–86)	68.7 (51–86)
Sex (Male:Female)	26:25	25:24
Performed surgery		
Cholecystectomy	11	
Acute ulceritis	8	
Hernia	5	
Thoracic stomach	2	
Healthy volunteers	24	

**Table 4:** Characteristics feature of PDAC patient cohort categorized according to the median expression of exosomal-CD44 (5.34) in plasma.

CD44 Plasma PDAC		Low	High	*p*-Value
Number		24	25	
Mean Age (in years [range])		65.8 (51–81)	72.0 (52–86)	**0.0336**
Sex	Female	13	11	0.48
	Male	11	14	
pT category	1	1	1	0.38
	2	5	7	
	3	10	4	
	4	1	1	
	Unknown/unresectable	7	12	
pN category	0	5	5	0.50
	1	6	5	
	2	6	3	
	Unknown/Inoperable	7	12	
Lymphatic invasion	L0	11	10	0.31
	L1	6	3	
	Unknown/Inoperable	7	12	
Venous invasion	V0	17	11	0.10
	V1	0	2	
	Unknown/Inoperable	7	12	
Perineural invasion	Pn0	5	5	0.35
	Pn1	12	8	
	Unknown/Inoperable	7	12	
R classification	R0	15	11	0.38
	R1	2	2	
	R2 (inoperable)	7	12	
Distant metastasis	M0	21	18	0.18
	M1	3	7	
UICC stage	I	1	4	0.21
	II	9	4	
	III	6	4	
	IV	3	7	
	Unknown	5	6	
Neoadjuvant treatment	Yes	4	5	0.76
	No	20	20	
Mean Preoperative CA19-9 (in u/mL [range])		157.0 (6.9–526.5)	168.4 (14.8–392.9)	0.8317

## Data Availability

The original contributions presented in this study are included in the article/Supplementary Materials. Further inquiries can be directed to the corresponding authors.

## Supplementary Materials

The following supporting information can be downloaded at https://www.mdpi.com/article/10.3390/cells15050411/s1; Figure S1: relative mRNA expression of CD44s and its variants according to tumor size (pT), nodal category (pN), metastasis status (pM) and lymphatic invasion status (L); Figure S2: relative mRNA expression of CD44s and its variants according to perineural invasion (Pn), venous invasion (V) and grading (G); Figure S3: relative mRNA expression of CD44s and its variants according to UICC staging; Figure S4: relative mRNA expression of CD44s and its variants according to PDAC patients that received treatment and patients who did not; Figure S5: Correlation of exo-CD44 with age and gender of PDAC patients; Figure S6: Correlation of exo-CD44 with systemic Bilirubin and leucocyte count of PDAC patients. Table S1: Characteristics feature of PDAC patient cohort categorized according to the median expression of relative mRNA expression CD44 (1.99) in LN.

## Abbreviations

The following abbreviations are used in this manuscript:

PDAC	Pancreatic ductal adenocarcinoma
LNs	Lymph nodes
Exo-CD44	Exosome-associated CD44
TCGA-PAAD	The Cancer Genome Atlas—Pancreatic Adenocarcinoma
CD44v	CD44 variant isoforms
qPCR	Quantitative polymerase chain reaction
GAPDH	glyceraldehyde 3-phosphate dehydrogenase
ELISA	Enzyme-Linked Immunosorbent Assay
EV	extracellular vesicle
PE	phycoerythrin
FITC	fluorescein isothiocyanate
APC	allophycocyanin
GO	Gene Ontology
KEGG	Kyoto Encyclopedia of Genes and Genomes
ROC	Receiver operating characteristic
GSEA	Gene set enrichment analysis
TME	Tumor microenvironment
HR	Hazard ratio
MaGIC	Molecular and Genomics Informatics Core
