# Supplementary material for: Immune Cell-Specific and Isoform-Selective Regulation of CD44 in Pancreatic Ductal Adenocarcinoma Links Lymph Node Variant Loss and Exosomal CD44 to Clinical Outcome in Pancreatic Ductal Adenocarcinoma
Source: Cells. 2026 Feb 27;15(5):411. doi: 10.3390/cells15050411 (PMC12984284; doi:10.3390/cells15050411)
Supplement: Supplementary file 1 [file cells-15-00411-s001.zip › cells-4162230-supplementary.pdf]

Figure S1

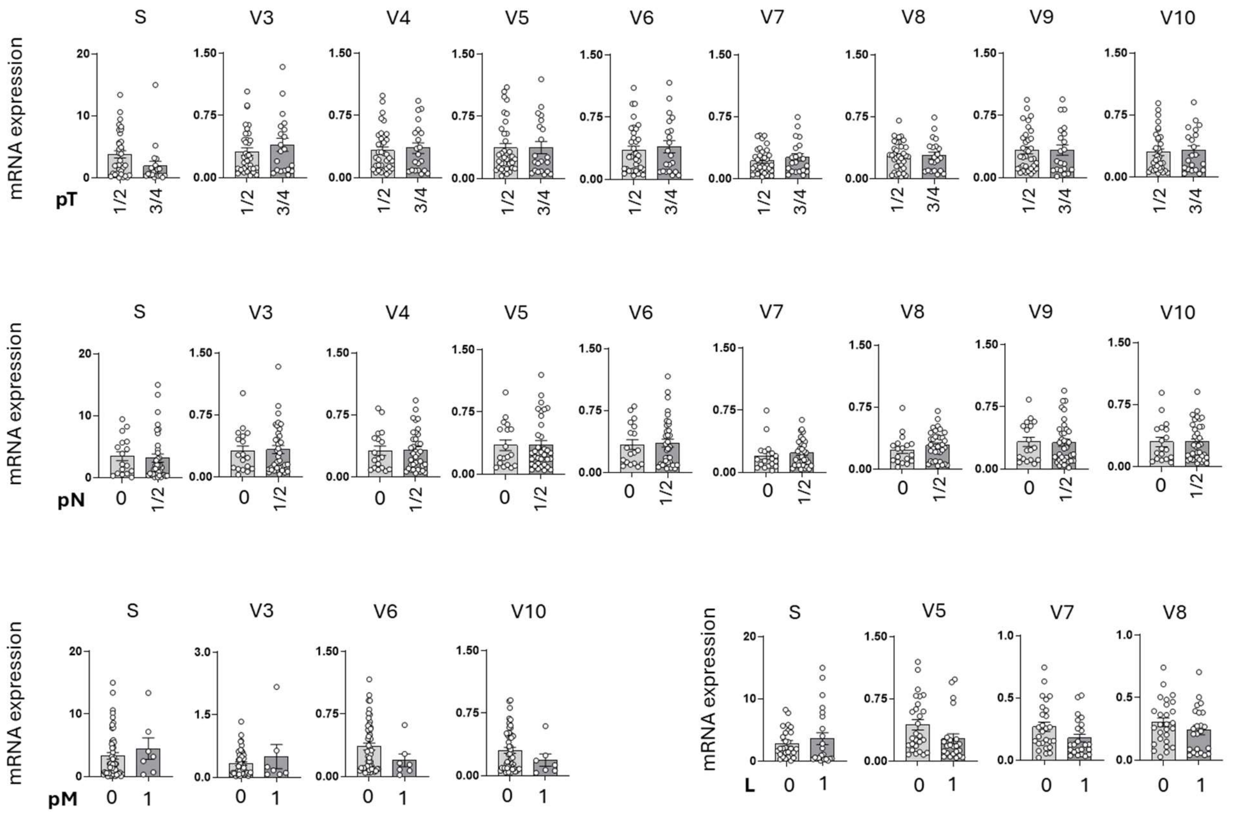

Figure S2

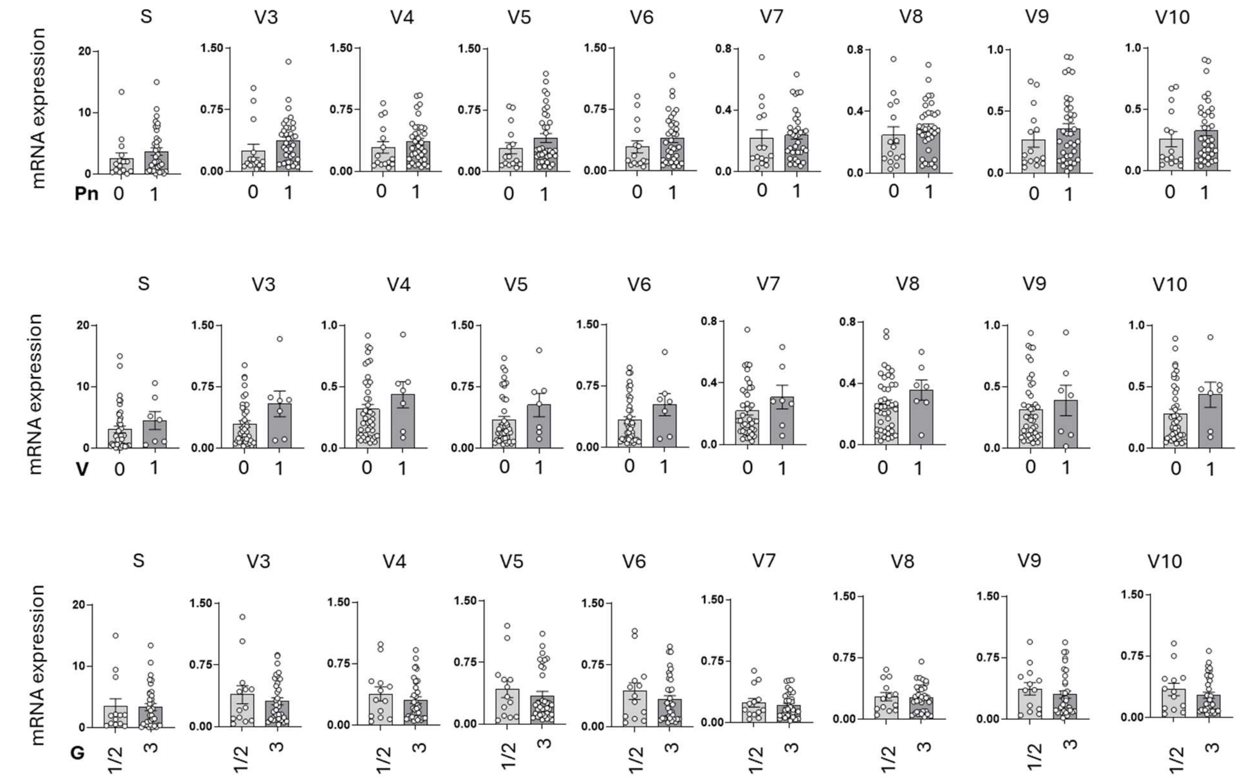

Figure S3

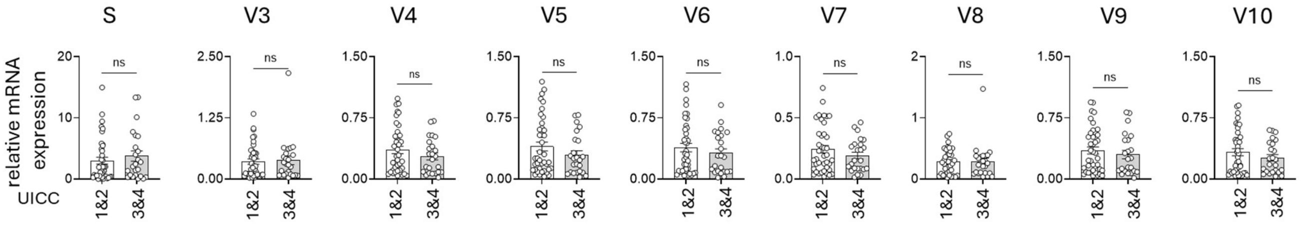

Figure S4

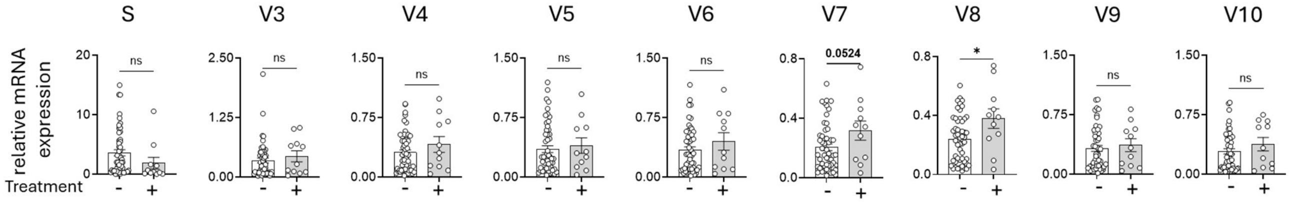

Figure S5

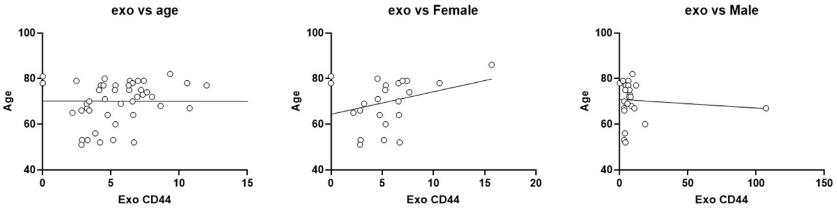

Figure S6

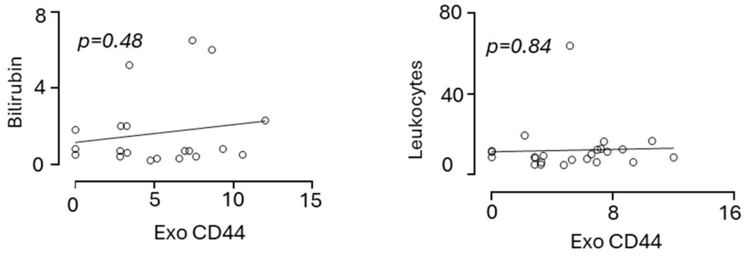

Supplementary Table S1: Characteristics feature of PDAC patient cohort categorized according to the median relative mRNA expression of CD44 (1.99) in LN

CD44 qPCR PDAC		Low	High	p-Value
Number		32	33	
Mean Age (in years [range])		68.3 (42-86)	68.5 (45-90)	0.4878
Sex	Female	13	18	0.26
	Male	19	15	
pT category	pT1	3	3	0.13
	pT2	6	15	
	pT3	11	5	
	pT4	1	0	
	Unknown/Inoperable	11	10	
pN category	pN0	5	7	0.77
	pN1,2	15	16	
	Unknown/Inoperable	12	10	
Lymphatic invasion	L0	9	14	0.46
	L1	12	9	
	Unknown/Inoperable	11	10	
Venous invasion	V0	10	20	0.28
	V1	3	2	
	Unknown/Inoperable	12	11	
Perineural invasion	Pn0	5	7	0.77
	Pn1	15	16	
	Unknown/Inoperable	12	10	

R-status	R0	23	23	0.08
	R1	2	1	
	R2/inoperable	7	9	
Grading	1	0	3	0.22
	2	6	3	
	3	16	19	
	unresectable/neoadjuvant	10	8	
Distant Metastasis	M0	30	26	0.08
	M1	2	7	
UICC stage	I	6	8	
	II	13	10	0.31
	III	9	5	
	IV	2	7	
	Unknown	2	3	
Neoadjuvant treatment	Yes	8	5	0.32
	No	24	28	
